# Can phenotypic data complement our understanding of antimycobacterial effects for drug combinations?

**DOI:** 10.1093/jac/dkz369

**Published:** 2019-08-25

**Authors:** Frank Kloprogge, Robert Hammond, Andrew Copas, Stephen H Gillespie, Oscar Della Pasqua

**Affiliations:** 1 Institute for Global Health, University College London, London, UK; 2 School of Medicine, University of St Andrews, St Andrews, UK; 3 Clinical Pharmacology and Therapeutics Group, School of Pharmacy, University College London, London, UK

## Abstract

**Objectives:**

To demonstrate how phenotypic cell viability data can provide insight into antimycobacterial effects for the isoniazid/rifampicin treatment backbone.

**Methods:**

Data from a *Mycobacterium komossense* hollow-fibre infection model comprising a growth control group, rifampicin at three different exposures (*C*_max_ = 0.14, 0.4 and 1.47 mg/L with *t*_½_ = 1.57 h and τ = 8 h) and rifampicin plus isoniazid (*C*_max_ rifampicin = 0.4 mg/L and *C*_max_ isoniazid = 1.2 mg/L with *t*_½_ = 1.57 h and τ = 8 h) were used for this investigation. A non-linear mixed-effects modelling approach was used to fit conventional cfu data, quantified using solid-agar plating. Phenotypic proportions of respiring (alive), respiring but with damaged cell membrane (injured) and ‘not respiring’ (dead) cells data were quantified using flow cytometry and Sytox Green™ (Sigma–Aldrich, UK) and resazurin sodium salt staining and fitted using a multinomial logistic regression model.

**Results:**

Isoniazid/rifampicin combination therapy displayed a decreasing overall antimicrobial effect with time ($\theta_{{\mathrm{Time}}_{1/2}}$ = 438 h) on cfu data, in contrast to rifampicin monotherapy where this trend was absent. In the presence of isoniazid a phenotype associated with cell injury was displayed, whereas with rifampicin monotherapy a pattern of phenotypic cell death was observed. Bacterial killing onset time on cfu data correlated negatively ($\theta_{{\mathrm{Time}}_{50}}$ = 28.9 h, $\theta_{{\mathrm{LAG}}_{{\mathrm{RIF}}_{50}}}$ = 0.132 mg/L) with rifampicin concentration up to 0.165 mg/L and this coincided with a positive relationship between rifampicin concentration and the probability of phenotypic cell death.

**Conclusions:**

Cell viability data provide structured information on the pharmacodynamic interaction between isoniazid and rifampicin that complements the understanding of the antibacillary effects of this mycobacterial treatment backbone.

## Introduction

Many infectious diseases require a combination of antimicrobial drugs to ensure complete pathogen clearance from the body. TB is a good example; it infects 9 million people worldwide each year and TB causes about 1.5 million deaths, which is more than any other infectious disease.^1^ Even standard treatment of drug-susceptible TB is complex, with daily oral administration of four antibiotics during the first 2 months, followed by daily oral administration of two antibiotics during a 4 month continuation phase.^2^

Current treatment evolved from a series of clinical trials over a period of 60 years.^3^ During this period, drugs and dosing regimens used in randomized controlled trial protocols were empirical with the evidence from one trial being used to plan the next. Consequently, little is known about how such combinations contribute to bacillary killing even though understanding this phenomenon in a quantitative manner would provide important insights into the activity of existing and novel drug combinations.

Recent efforts to shorten the treatment of drug-susceptible disease have shown that the experimental regimens were not non-inferior compared with standard care^4^^,^^5^ and consequently did not lead to changes in treatment guidelines. These findings have prompted further evaluation of the underlying drug interactions and dosing regimens required for combination therapy. In this context, modelling and simulation concepts can provide insight and guidance towards drug and dose selection for treatment combinations for TB treatment. Using pharmacokinetic/pharmacodynamic (PKPD) modelling, for example, one may be able to characterize bacterial clearance using killing rate constants. This is crucial to infer maximum killing, which can subsequently be used to more accurately predict the time required to achieve complete eradication of the bacterial load.

Predicting the bacillary killing rate remains challenging as treatment of TB requires four antibiotic drugs in different combinations over time.^2^ Moreover, it is important to evaluate the impact of pharmacokinetic characteristics of drug combinations in non-clinical protocols to evaluate total antitubercular activity. Most animal models do not provide exposure profiles that mimic human exposure for known drugs; animal models are also less flexible when evaluating new drug combinations, as exposure ratios change over time due to species-specific differences in drug clearance. In contrast, some of these limitations can be overcome in a hollow-fibre system where multiple experiments can be performed more rapidly.^6^

Key characteristics of bacillary clearance for anti-TB drugs have been identified *in vitro* using the hollow-fibre infection model, such as a variable antimycobacterial effect of isoniazid over the course of treatment,^7^ and these findings correspond with *in vivo* antitubercular activity.^8^ These findings suggest not only that drugs may act on different parts of a cell, but that infections consist of different subpopulations of cells, which can have varying susceptibility to drugs. Most *in vitro* anti-tuberculosis studies use cfu and these data may not reflect the activity against susceptible, resistant or non-replicating bacterial subpopulations. Hence, attention is required to ensure accurate translation and interpretation of the results from such experimental protocols. Quantitative information on the number of respiring (alive) versus ‘not respiring’ (dead) cells and cells with damaged cell membranes (injured) or intact cell membranes using calcein violet and Sytox Green™ staining methods can be a valuable tool and may provide insight into bacterial fitness.^9^

Parameterization of pharmacokinetic and bacillary killing data in PKPD models provides the possibility to interpolate and extrapolate bacillary clearance under different scenarios with the computer. Simulations to evaluate the appearance of persistent and tolerant bacterial subpopulations over the course of a treatment can provide crucial information.^10^^,^^11^ A variety of PKPD models have been developed to support antimicrobial drug combination research^12^^,^^13^ using *in vitro*,^14^ pre-clinical^15^^,^^16^ and clinical data.^17^ However, as for many other infectious diseases,^18–22^ rather complex model structures that include susceptible, resistant or non-replicating subpopulations have been used to parameterize bacillary clearance characteristics based on cfu data only.^14^ The concept of parameterizing the appearance of these subpopulations, i.e. bacterial fitness, into PKPD models is valid although it has to be data driven,^23^ for example using data on bacterial fitness.

The aim of this investigation was therefore to demonstrate the potential of integrating both cell viability and cfu data in two separate PKPD models to complement the understanding of anti-bacillary effects of drug combinations using isoniazid/rifampicin and *Mycobacterium komossense* as a paradigm.

## Materials and methods

### Hollow-fibre model of infection

Isoniazid and rifampicin pharmacokinetic profiles in lung lesion homogenate, mimicking 600 mg and 300 mg daily oral doses of rifampicin (450 mg for patients <50 kg in body weight) and isoniazid, respectively, were generated in the hollow-fibre model of infection. Drug levels generated in the hollow-fibre model of infection were also adjusted for protein binding, namely 42% for isoniazid and 83% for rifampicin (Table 1).^24^ To ensure pharmacokinetic characteristics of the drugs were matched to the *M. komossense* growth characteristics, *T*_max_ and elimination *t*_½_ were divided by three to adjust for the known differences in pathogen life cycle, i.e. 8 h versus 24 h for *M. komossense* and *Mycobacterium tuberculosis,* respectively.

**Table 1.** Summary of pharmacokinetic settings for the hollow-fibre model of infection experiments

		Rifampicin			
Parameter	Growth curve	*C*_max_ = 0.14 mg/L	*C*_max_ = 0.4 mg/L	*C*_max_ = 1.47 mg/L	Rifampicin *C*_max_ = 0.4 mg/L + isoniazid *C*_max_ = 1.2 mg/L
Rifampicin C_T=2 min_ (mg/L)	—	0.00651	0.0186	0.0686	0.0186
Rifampicin *C*_max_ (mg/L)	—	0.14	0.400	1.47	0.400
Rifampicin AUC (mg·h/L)	—	0.317	0.905	3.34	0.905
Isoniazid *C*_max_ (mg/L)	—	—	—	—	1.20
Isoniazid AUC (mg·h/L)	—	—	—	—	2.73
*T*_max_ (h)	—	0.717	0.717	0.717	0.717
*t*_½_ (h)	—	1.57	1.57	1.57	1.57
τ (h)	—	8	8	8	8

C_T=2 min_, predicted concentration 2 min after dosing; *C*_max_, predicted maximum concentration; AUC, predicted AUC at steady-state; *T*_max_, predicted time at which maximum concentration occurs; *t*_½_, predicted elimination *t*_½_; τ, dosing interval or time between dose administration.

A web application (https://pkpdia.shinyapps.io/hfs_app/) was used to convert adjusted secondary pharmacokinetic parameter estimates, *C*_max_, *T*_max_ and *t*_½_, into pump settings at a system volume of 133 mL (central reservoir, 75 mL; intracapillary space and tubing, 44 mL; and extracapillary space, 14 mL). Prior to *T*_max_ being reached, drugs were infused using a zero-order process into the central reservoir; thereafter flow rates between the diluent and central reservoir and central reservoir and elimination reservoir were set at an identical rate.

*M. komossense* (ATCC 33013) was incubated with Middlebrook 7H9 (Fluka) and 0.05% Tween (Sigma–Aldrich, UK) in sealed 50 mL tubes (Falcon, Corning, USA) at 30°C. This was done 3–5 days prior to inoculation of the hollow-fibre model of infection until OD_600_ > 0.1. The hollow-fibre infection model was run at 30°C. The rifampicin experiments (at *C*_max_ = 0.14, 0.4 and 1.47 mg/L with *t*_½_ = 1.57 h and τ = 8 h) and the rifampicin/isoniazid experiment (at *C*_max_ rifampicin = 0.4 mg/L and *C*_max_ isoniazid = 1.2 mg/L with *t*_½_ = 1.57 h and τ = 8 h) lasted for 7 days and the control experiments lasted for 10 days to ensure adequate characterization of the maximum carrying capacity. All experiments were performed as single runs and samples for bacterial load quantification and assessment of cell viability were taken every 24 h on weekdays. Drug concentrations in the hollow-fibre medium were not measured during the experiments.

### Viable count measurement

Viable counts from daily samples from the hollow-fibre infection model were determined by cfu counts on Middlebrook 7H11 agar (Sigma –Aldrich, UK) as described previously.

### Assessment of cell viability

*M. komossense* viability was assessed using Sytox Green™ (Sigma–Aldrich) and resazurin sodium salt (alamar blue) (Sigma–Aldrich). Cultures were stained with resazurin at 0.01% solution overnight (16 h) in the dark and in the last hour of the incubation period Sytox Green™ was introduced at 20 μM. Quantification was performed by flow cytometry using a Millipore Guava easyCyte™ HT system at 488 nm (blue light) and collected signal at 525/30 nm and 690/50 nm. Samples were loaded into a flat-bottomed 96-well plate (Nunc, Thermo Fisher, Denmark).

### Modelling of cfu counts

A compartmental model using cfu data was fitted using NONMEM 7.3 on a Windows 10 operating system. Data were transformed into logarithm base 10 and minus twice the log likelihood of the data was used as objective function value (OFV). ADVAN9 and the FOCE-I method was used for estimation. Mean rifampicin population predictions were used as input for subsequent PKPD analysis.^25^ Inclusion of one degree of freedom to a nested hierarchical model was considered to improve the model’s ability to fit the data statistically if a drop in OFV of at least 3.84 (*P *=* *0.05) was achieved. Assessment of model performance was further supported by goodness-of-fit diagnostics.

Baseline bacterial load at experiment level (*P_i_*) was estimated using typical baseline bacterial load (*θ_TV_*) and a deviation from the typical baseline bacterial load for the rifampicin plus isoniazid experiment (*COV*) with a residual error term (*ε*) (Eq. 1).

(1) $$P_{i}=10^{\theta_{\mathrm{TV}}\times \left(1+\mathrm{COV}\right)+\varepsilon }$$

Data from the growth control experiment was used to develop a log growth model (Eq. 2) using the parameters net growth ($\theta_{k_{\mathrm{net}}}$) and maximum carrying capacity ($\theta_{{\mathrm{cfu}}_{\mathrm{MAX}}}$).

(2) $$\frac{\mathrm{dcfu}}{\mathrm{dt}}=\theta_{k_{\mathrm{net}}}\times \mathrm{cfu}\times \log\left(\frac{10^{\theta_{{\mathrm{cfu}}_{\mathrm{MAX}}}}}{\mathrm{cfu}}\right)$$

Data from the rifampicin therapy experiments were subsequently integrated into the analysis to describe the antimicrobial effect of rifampicin (*E*; Eq. 3). Drug effects were parameterized in terms of a maximum drug effect ($\theta_{E_{\mathrm{MAX}}}$) and potency, i.e. the concentration at which half-maximum inhibition ($\theta_{{\mathrm{IC}}_{50}}$) is achieved, where *C* is the predicted mean rifampicin concentration in the medium.

(3) $$E=\frac{\theta_{E_{\mathrm{MAX}}}\times C}{\theta_{{\mathrm{IC}}_{50}}+C}$$

The onset time of the rifampicin antimicrobial effect was rifampicin concentration-dependent (*E_LAG_*; Eq. 4) and this was parameterized by the time at which half-maximum inhibition ($\theta_{{\mathrm{Time}}_{50}}$) was achieved, which is dependent on the rifampicin concentration that realizes half-maximum delay ($\theta_{{\mathrm{LAG}}_{{\mathrm{RIF}}_{50}}}$). *C* is the predicted mean rifampicin concentration and Time is observed time in hours.

(4) $$E_{\mathrm{LAG}}=\frac{{\mathrm{Time}}^{20}}{{\mathrm{Time}}^{20}+{\left(\theta_{{\mathrm{Time}}_{50}}(1-\frac{C^{20}}{C^{20}+\theta_{{\mathrm{LAG}}_{{\mathrm{RIF}}_{50}}}})\right)}^{20}}$$

Parameters describing growth characteristics (Eq. 2), rifampicin drug effect (Eq. 3) and delay in rifampicin effect (Eq. 4) were fixed when data from the isoniazid plus rifampicin experiment were included for evaluation of the isoniazid/rifampicin interaction effect. The variable antimycobacterial effect over the course of treatment ($E_{\mathrm{Cease}}$), after isoniazid inclusion in the rifampicin experiment, was parameterized using an exponential model with the parameter time to half-maximum drug effect inhibition ($\theta_{{\mathrm{Time}}_{1/2}}$; Eq. 5).

(5) $$E_{\mathrm{Cease}}=1-(1-e^{-\mathrm{Time}\frac{\log(2)}{\theta_{{\mathrm{Time}}_{1/2}}}})$$

Change in cfu over time was consequently parameterized as (Eq. 6):

(6) $$\frac{\mathrm{dcfu}}{\mathrm{dt}}={(\theta }_{k_{\mathrm{net}}}-(E\times E_{\mathrm{LAG}}\times E_{\mathrm{Cease}}))\times \mathrm{cfu}\times \log\left(\frac{10^{\theta_{{\mathrm{cfu}}_{\mathrm{MAX}}}}}{\mathrm{cfu}}\right)$$

Residual variability (*ε*) departing from model predictions (*IPRED*) to observations (*y*) was additive on two-sided log_10_-transformed data (Eq. 7):

(7) y=IPRED+ε

Model parameters have been reported in their original form in the Supplementary data (available at *JAC* Online) and relevant model parameters have been converted from nmol/L to mg/L and reported in the manuscript tables and figures.

### Cell viability data parameterization

An unordered multinomial response model was developed to parameterize *M. komossense* viability data using the software package NONMEM 7.3 on a Windows 10 operating system. The FOCE-I method was used for estimations and mean rifampicin population predictions were used as input for subsequent PKPD analysis. Goodness-of-fit diagnostics and the OFV, defined as minus twice the log likelihood of the summary statistics data, was used to discriminate between hierarchical models. A drop in OFV of at least 3.84 (*P *=* *0.05) after inclusion of one degree of freedom was considered statistically significant and reflected an improvement of the model.

Baseline data samples were taken 2 min after drug was added to the hollow-fibre infection model. Treatment effect was described using a logistic regression model, in which the log ratio of the probabilities of being alive to injured (Eq. 8) and the log ratio of the probabilities of being dead to injured (Eq. 9) were derived. The relationship between the proportion of alive, injured and dead bacteria at baseline in the growth curve, low, medium and high rifampicin experiment (*j*) and baseline rifampicin levels (predicted at 2 min after start of the experiment) were estimated first. Serial samples from the growth curve and the low, medium and high rifampicin experiment were used during the analysis to characterize the effect of rifampicin exposure (AUC_0–24_) over time on the log ratio of the probabilities of being alive to injured (Eq. 8) and the log ratio of the probabilities of being dead to injured (Eq. 9). Subsequently, serial samples from the isoniazid plus rifampicin experiment were analysed to characterize drug–drug interaction under the assumption of an additive covariate effect on the log ratio of the probabilities of being alive to injured (Eq. 8) and the log ratio of the probabilities of being dead to injured (Eq. 9), where Pr is probability.

(8) $$\log\left(\frac{\mathrm{Pr}\;(\mathrm{Alive})}{\mathrm{Pr}\;(\mathrm{Injured})}\right)=\theta \left(1\right)+{\left(\theta \left(2\right)+(\theta \left(3\right)\times C_{\mathrm{Time}=2\;\mathrm{minutes}})\right)}_{j\neq \mathrm{growth}\;\mathrm{control}}+\left({\left(\theta \left(4\right)+(\theta \left(5\right)\times {\mathrm{AUC}}_{0-24})\right)}_{j=\mathrm{rifampicin}}\times {\theta \left(6\right)}_{j=\mathrm{isoniazid}+\mathrm{rifampicin}}\right)\times \mathrm{Time}$$

(9) $$\log\left(\frac{\mathrm{Pr}\;(\mathrm{Dead})}{\mathrm{Pr}\;(\mathrm{Injured})}\right)=\theta \left(7\right)+\left(\theta \left(8\right)\times C_{\mathrm{Time}=2\;\mathrm{minutes}}\right)+\left({\left(\theta \left(9\right)+(\theta \left(10\right)\times {\mathrm{AUC}}_{0-24})\right)}_{j=\mathrm{rifampicin}}\times {\theta \left(11\right)}_{j=\mathrm{isoniazid}+\mathrm{rifampicin}}\right)\times \mathrm{Time}$$

Probability of cells in a sample being alive (Eq. 10), dead (Eq. 11) or injured (Eq. 12) can be derived as follow:

(10) $$\mathrm{Pr}\;(\mathrm{Alive})=\frac{\frac{\mathrm{Pr}\;(\mathrm{Alive})}{\mathrm{Pr}\;(\mathrm{Injured})}}{1+\frac{\mathrm{Pr}\;(\mathrm{Alive})}{\mathrm{Pr}\;(\mathrm{Injured})}+\frac{\mathrm{Pr}\;(\mathrm{Dead})}{\mathrm{Pr}\;(\mathrm{Injured})}}$$

(11) $$\mathrm{Pr}\;(\mathrm{Dead})=\frac{\frac{\mathrm{Pr}\;(\mathrm{Dead})}{\mathrm{Pr}\;(\mathrm{Injured})}}{1+\frac{\mathrm{Pr}\;(\mathrm{Alive})}{\mathrm{Pr}\;(\mathrm{Injured})}+\frac{\mathrm{Pr}\;(\mathrm{Dead})}{\mathrm{Pr}\;(\mathrm{Injured})}}$$

(12) Pr (Injured)=1-Pr (Alive)-Pr (Dead)

Considering that the summary statistics at each sampling timepoint were analysed and not the state of each individual bacterium, residual variability (*ε*), departing from model-predicted probabilities (*IPRED*) to the observed proportions (*y*) was additive in a logit transformation (Eqs 13–15). Residual variability was described as follow:

(13) $$\varphi_{\mathrm{TV}}=\log\;\left(\frac{\mathrm{IPRED}}{1-\mathrm{IPRED}}\right)$$

(14) $$\varphi =\varphi_{\mathrm{TV}}+\varepsilon$$

(15) $$y=\frac{e^{\varphi }}{1+e^{\varphi }}$$

Model parameters have been reported in their original form in the Supplementary data and rifampicin concentrations were converted from nmol/L to mg/L for presentation in the manuscript Tables and Figures.

## Results

### cfu modelling

**Figure 1.**
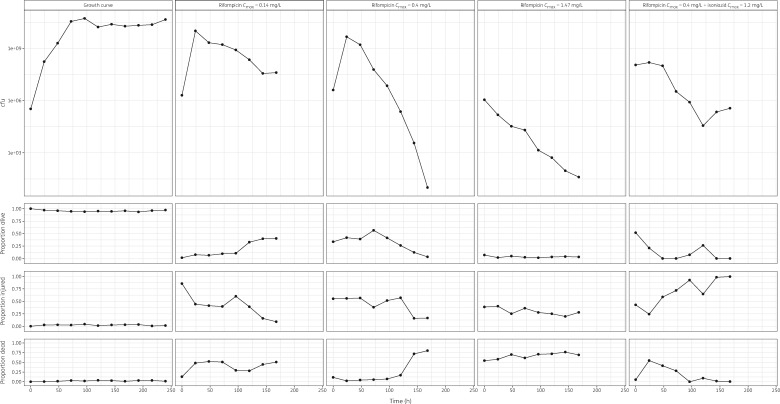
Visual representation of raw cfu–time data (top panels) and proportions of alive/injured/dead–time data (bottom three panels) for a growth curve experiment, a low (*C*_max_ = 0.14 mg/L), medium (*C*_max_ = 0.4 mg/L) and high (*C*_max_ = 1.47 mg/L) rifampicin exposure experiment and a medium rifampicin exposure plus isoniazid experiment (*C*_max rifampicin_ = 0.4 mg/L and *C*_max isoniazid_ = 1.2 mg/L). Dots represent observations and black lines are connecting lines.

**Figure 2.**
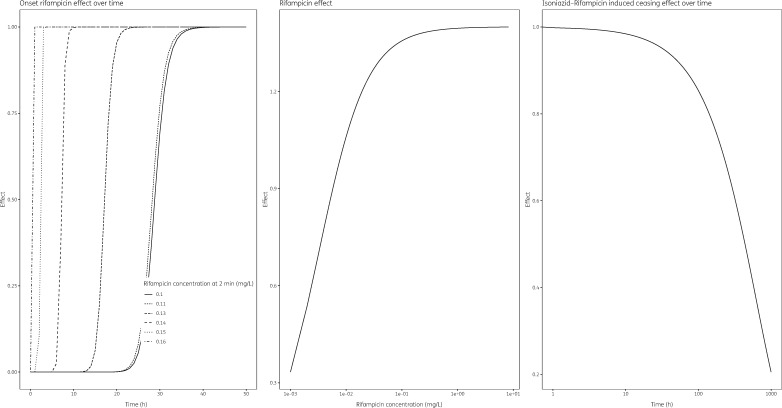
Visualization of drug effect characteristics in the differential equation model on cfu–time data. Rifampicin concentration-dependent effect onset (left panel), rifampicin effect (middle panel), and total isoniazid/rifampicin ceasing effect over time (right panel).

cfu data (Figure 1) were fitted using an empirical turnover model (Table S1, Figures S1 and S2) and the antibacterial effect of rifampicin was parameterized as an additive drug effect with IC_50_ at 0.00320 mg/L and *E*_max_ at 1.4. Rifampicin levels between 0.000823 and 0.411 mg/L resulted in substantial changes in bacterial killing, whereas rifampicin levels above 0.823 mg/L did not (Figure 2).

The predicted delay to the onset of rifampicin effect was dependent on rifampicin levels with $\theta_{{\mathrm{LAG}}_{{\mathrm{RIF}}_{50}}}$ at 0.132 mg/L and $\theta_{{\mathrm{Time}}_{50}}$ at 28.9 h (Table S1). Consequently, rifampicin levels of 0.165 mg/L or higher caused an almost immediate antibacterial effect whereas concentrations lower than 0.165 mg/L also showed antibacterial activity but the effect is predicted to occur at a later time after start of drug administration (up to ∼30 h) (Figure 2).

Inclusion of isoniazid in combination with rifampicin caused the overall antibacterial effect to decrease with time. At 438 h post-start of the experiment, half-maximum drug effect inhibition was reached (Table S1, Figure 2).

### Cell viability data

**Figure 3.**
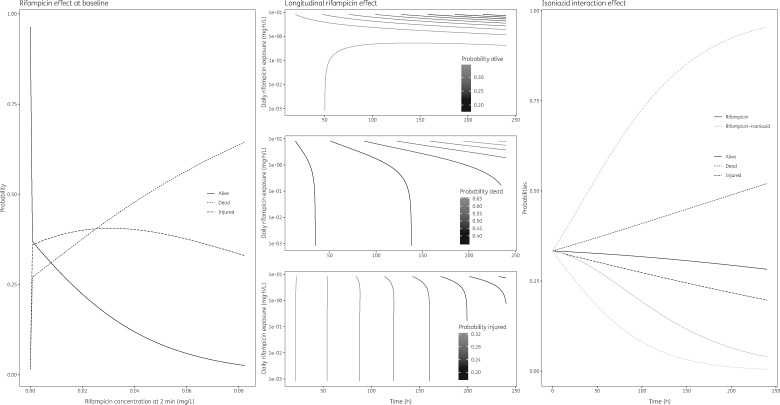
Visualization of drug effect characteristics in the multinomial regression model on bacterial viability–time data. Rifampicin-induced effect at baseline (left panel), rifampicin effect over time (middle panel) and total isoniazid/rifampicin effect over time (right panel).

Cell viability data (Figure 1) were fitted using an unordered categorical regression model (Table S2, Figures S3 and S4) and rifampicin exposure positively correlated with the proportion of dead cells over time (Figure 3). On the other hand, the probability of cells being injured or alive negatively correlated with rifampicin exposure over time (Figure 3).

Rifampicin also displayed distinct characteristics 2 min after the start of the experiment. Whilst a negligible proportion of the cells at baseline were injured or dead in the growth control experiment the proportion of alive cells dropped with increasing rifampicin concentration (Figure 3). Conversely, the proportion of dead cells increased with increasing rifampicin levels and the proportion of injured cells displayed an inverse quadratic relationship with rifampicin levels (Figure 3).

Other than when rifampicin was studied alone, inclusion of isoniazid in combination with rifampicin resulted in a decreased probability of alive and dead cells over time and an increased probability of injured cells over time (Figure 3).

## Discussion

Understanding bacillary killing characteristics for antimicrobial drug combinations is both important and challenging. Historically, drug combinations, such as for the treatment of TB, have been selected based on empirical evidence and such a setting creates a problem when one wants to identify novel combinations for both susceptible- and drug-resistant TB. The hollow-fibre infection model combined with live/dead staining techniques makes it possible to not only explore the antibacterial activity whilst taking into account the impact of different pharmacokinetic profiles, but also to integrate it with evolving technologies, which enable the characterization of phenotypes/subpopulations. Such an experimental setting offers a unique opportunity to evaluate antibacillary effects in a parametric manner, yielding estimates of antibacillary effects that can be compared across different compounds in a dose-independent manner. Such data provide the basis for the selection of compounds as well as further insight into the optimization of doses and dosing regimens.

Due to the complexity of bacillary killing data, often displaying biphasic elimination, assumptions about formation of susceptible, resistant or dormant bacterial subpopulations over time have been made^14^^,^^17–22^ without adequate data being available to support these hypotheses, resulting in identifiability problems.^23^ Parameterization of staining data from bacterial samples such as proportion of alive/injured/dead cells over the treatment course in a logistic regression model can be a data-driven additional source of information to previously described models,^14^ providing a comprehensive insight into antimicrobial drug effects. However, for parameterization of this type of data a multinomial unordered logistic regression model should be used.

The rifampicin-driven bacillary killing displayed in the cfu data (Figure 2) coincided with the findings seen in viability data, in that the probability of cells being dead correlated positively with rifampicin AUC_0-24_ (Figure 3). The delayed onset of rifampicin-induced bacillary killing seen in the conventional cfu data (Figure 2) coincided with the considerable proportions of injured and dead cells at baseline seen in viability data (Figure 3), in that increasing early rifampicin concentrations correlate negatively with time to bacillary killing onset and positively with the proportion of dead cells.

The delayed onset of rifampicin killing has not been previously reported, although published *in vivo*-mimicking hollow-fibre infection models for rifampicin used *M. tuberculosis*^26–29^ and the experiments presented here were performed with a different strain (*M. komossense*). Moreover, previous research simulated free rifampicin profiles in blood^26–29^ and these levels are higher compared with the free lung lesion homogenate exposures simulated in this study. The higher rifampicin levels in published research may explain the direct onset of rifampicin-driven antimicrobial effect.

The addition of isoniazid reduced rifampicin bacillary killing over time in the cfu data (Figure 2) and this coincided with an increased probability of injured cells over time at the cost of decreased probability of both alive and dead cells (Figure 3). The change in proportions of alive/injured/dead cells overlaps with previously reported ceasing isoniazid effect over the course of the treatment, which has been confirmed *in vivo* in patients and *in vitro* in a hollow-fibre infection model with *M. tuberculosis*.^7^^,^^8^ The latter study attributed this to the emergence of genotypic resistance.^7^ However, both experiments reported their findings based on isoniazid monotherapy and to the best of our knowledge this research describes the isoniazid–rifampicin interaction for the first time in an *in vitro* hollow-fibre infection model. These experiments should be replicated using *M. tuberculosis* to confirm the observations at varying isoniazid concentrations and higher rifampicin concentrations than currently tested, alone and in combination. This will allow us to identify target exposure for the isoniazid/rifampicin combination that maximizes bacterial killing whilst minimizing the ceasing of the killing effect over time, which is key in light of current efforts to increase clinical rifampicin dosing.^30^

The presented findings remain descriptive and are limited by the small sample size, and the impact of different experimental protocols has not been evaluated. Model parameterization is data driven and primarily aimed at describing the available data. The viability of cells in the hollow-fibre model of infection may need to be further characterized and different drugs and dosing regimens need to be tested to ensure the generalizability of the findings.

In summary, with the use of mathematical models becoming increasingly more common, we have shown that the evolution of bacterial viability over time can be fitted using empirical data-driven models. This type of data analysis, in addition to bacillary killing characteristics, can improve the understanding of the interaction of the moieties in a drug combination and provide the basis for hypothesis generation regarding treatment response in animal models and in humans. From a clinical microbiology perspective, addition of isoniazid reduced rifampicin bacillary killing over time in cfu data, coinciding with increased proportions of injured cells. Confirmation of these results using *M. tuberculosis* is required to establish the generalizability of the effect across different strains.

## Supplementary Material

dkz369_Supplementary_Data
