# Supplementary material for: Can phenotypic data complement our understanding of antimycobacterial effects for drug combinations?
Source: J Antimicrob Chemother. 2019 Aug 25;74(12):3530–6. doi: 10.1093/jac/dkz369 (PMC6857198; doi:10.1093/jac/dkz369)
Supplement: dkz369_Supplementary_Data [file dkz369_supplementary_data.docx]

SUPPLEMENTARY MATERIAL: Can phenotypic data complement our understanding of anti-mycobacterial effects for drug combinations?

Table of Contents

[Colony Forming Units data 2](#_Toc16591034)

[Table S1: Parameters from the turnover model on Colony Forming Units data 2](#_Toc16591035)

[Figure S1: Goodness of fit plots for the turnover model 3](#_Toc16591036)

[Figure S2: Individual data fits for the turnover model 4](#_Toc16591037)

[Cell viability data 5](#_Toc16591038)

[Table S2: Parameter estimates from the unordered multinomial response model 5](#_Toc16591039)

[Figure S3: Goodness of fit plots for the unordered multinomial response model. 6](#_Toc16591040)

[Figure S4: Individual data fits for the unordered multinomial response model. 7](#_Toc16591041)

# Colony Forming Units data

## Table S1: Parameters from the turnover model on Colony Forming Units data

|  | Estimate | %RSE |
| --- | --- | --- |
| Baseline CFU (10^) | 6.17 | 3.34 |
| knet (hr-1) | 0.04 | 6.26 |
| CFUMAX | 10.50 | 0.88 |
| IC50 (nmol/l) | 3.89 | 163 |
| EMAX | 1.40 | 1.36 |
| Time50 (hr) | 28.9 | 9.13 |
| LagRif50 (nmol/l) | 161 | 846 |
| INH on baseline CFU | 0.31 | 16.3 |
| T1/2 INH (hr) | 438 | 0.06 |
| OMEGA EC50 | 5.20 | 112 |
| OMEGA ECLAG50 | 3.93 | 2360 |
| Additive residual variability | 0.39 | 2.50 |

##

## Figure S1: Goodness of fit plots for the turnover model


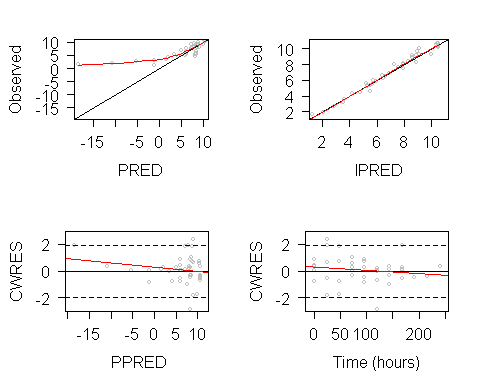


Popupation (PRED) and individual (IPRED) predictions against observations, and conditonal weighted residuals (CWRES) versus PRED and time after start of the experiment. Dots represent observations, the black line represents the line of unity and the red line represents the Local Polynomial Regression Fitting.

## Figure S2: Individual data fits for the turnover model


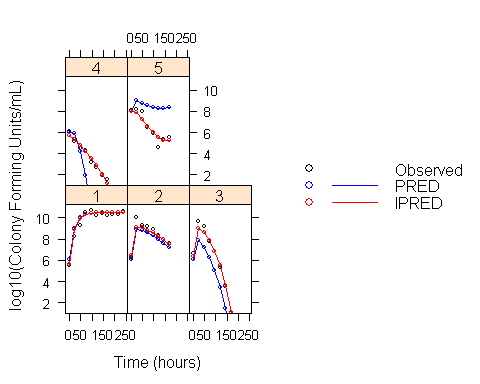


Population (PRED) and individual (IPRED) predictions of the observed CFU data with the turnover model. 1: Growth curve, 2: Rifampicin C_MAX_ = 0.14 mg/l, 3: Rifampicin C_MAX_ = 0.4 mg/l, 4: Rifampicin C_MAX_ = 1.47 mg/l, and 5: Rifampicin C_MAX_ = 0.4 mg/l & isoniazid C_MAX_ = 1.2 mg/l.

# Cell viability data

## Table S2: Parameter estimates from the unordered multinomial response model

|  | Estimate | Std. Error |
| --- | --- | --- |
| THETA1 | 3.86e+00 | 1.71e-01 |
| THETA2 | -3.81e+00 | 6.17e-01 |
| THETA3 | -2.61e-02 | 1.03e-02 |
| THETA4 | -3.00e-07 | 6.00e-07 |
| THETA5 | 2.34e-03 | 6.06e-03 |
| THETA6 | -8.84e+00 | 2.74e+01 |
| THETA7 | 9.65e-03 | 4.35e-03 |
| THETA8 | -2.96e-01 | 2.73e-01 |
| THETA9 | 2.00e-07 | 3.00e-07 |
| THETA10 | 3.35e-03 | 3.04e-03 |
| THETA11 | -5.41e+00 | 3.80e+00 |
| Additive | 7.19e-01 | 2.04e-01 |

##

## Figure S3: Goodness of fit plots for the unordered multinomial response model.


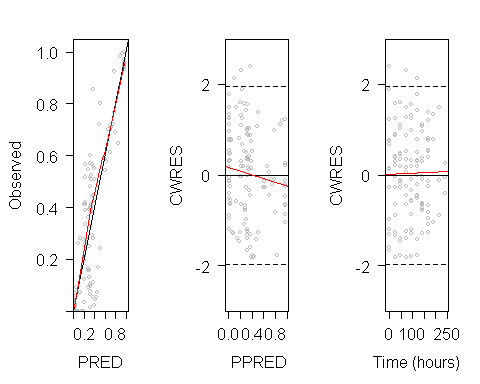


Model predictions (PRED) against observed proportions, and conditonal weighted residuals (CWRES) versus PRED and time after start of the experiment. Dots represent observations, the black line represents the line of unity and the red line represents the Local Polynomial Regression Fitting.

## Figure S4: Individual data fits for the unordered multinomial response model.


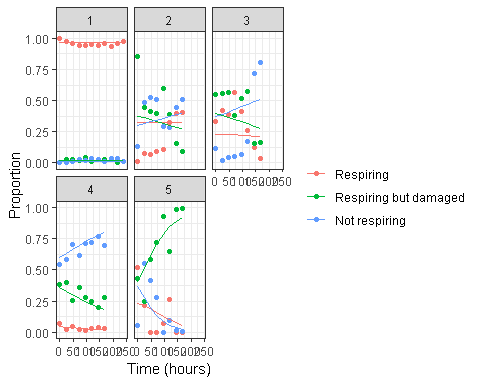


Model predictions (blue, green and red lines) of the observed proportional data (blue, green and red dots). 1: Growth curve, 2: Rifampicin C_MAX_ = 0.14 mg/l, 3: Rifampicin C_MAX_ = 0.4 mg/l, 4: Rifampicin C_MAX_ = 1.47 mg/l, and 5: Rifampicin C_MAX_ = 0.4 mg/l & isoniazid C_MAX_ = 1.2 mg/l. Model predictions for the Rifampicin C_MAX_ = 0.14 mg/l and Rifampicin C_MAX_ = 0.4 mg/l experiment were less accurate when compared to the model predictions for the growth curve, Rifampicin C_MAX_ = 0.14 mg/l, and Rifampicin C_MAX_ = 0.4 mg/l & isoniazid experiment due to the noise in the data.
